# Quercetin suppresses immune cell accumulation and improves mitochondrial gene expression in adipose tissue of diet‐induced obese mice

**DOI:** 10.1002/mnfr.201500595

**Published:** 2015-11-24

**Authors:** Masuko Kobori, Yumiko Takahashi, Mutsumi Sakurai, Yukari Akimoto, Tojiro Tsushida, Hideaki Oike, Katsunari Ippoushi

**Affiliations:** ^1^ National Food Research InstituteNational Agriculture and Food Research OrganizationTsukubaIbarakiJapan; ^2^ Department of Food BusinessSchool of FoodAgricultural and Environmental SciencesMiyagi UniversitySendaiMiyagiJapan

**Keywords:** Adipose tissue, Inflammation, Obesity, Oxidative stress, Quercetin

## Abstract

**Scope:**

To examine the effect of dietary quercetin on the function of epididymal adipose tissue (EAT) in Western diet‐induced obese mice.

**Methods and results:**

C57BL/6J mice were fed a control diet; a Western diet high in fat, cholesterol, and sucrose; or the same Western diet containing 0.05% quercetin for 18 weeks. Supplementation with quercetin suppressed the increase in the number of macrophages, the decrease in the ratio of CD4^+^ to CD8^+^ T cells in EAT, and the elevation of plasma leptin and tumor necrosis factor α levels in mice fed the Western diet. Comprehensive gene expression analysis revealed that quercetin suppressed gene expression associated with the accumulation and activation of immune cells, including macrophages and lymphocytes in EAT. It also improved the expression of the oxidative stress‐sensitive transcription factor NFκB, NADPH oxidases, and antioxidant enzymes. Quercetin markedly increased gene expression associated with mitochondrial oxidative phosphorylation and mitochondrial DNA content.

**Conclusion:**

Quercetin most likely universally suppresses the accumulation and activation of immune cells, including antiinflammatory cells, whereas it specifically increased gene expression associated with mitochondrial oxidative phosphorylation. Suppression of oxidative stress and NFκB activity likely contributed to the prevention of the accumulation and activation of immune cells and resulting chronic inflammation.

**Abbreviations**
AMPK: adenosine monophosphate‐activated protein kinase
Cat: catalase
EAT: epididymal adipose tissue
GEO: gene expression omnibus
Gpx1: glutathione peroxidase 1
HO‐1: oxygenase 1
Lox‐1: lectin‐like oxidized low‐density lipoprotein receptor‐1
MDA: malondialdehyde
MHCII: class II major histocompatibility complex
mtDNA: mitochondrial DNA
Nrf2: nuclear factor (erythroid derived 2)‐like 2
PGC‐1**α**: peroxisome proliferative‐activated receptor gamma, coactivator 1 alpha
Sod1: superoxide dismutase 1
SRM: selected reaction monitoring
TNF**α**: tumor necrosis factor α
VAT: visceral adipose tissue

## 1 Introduction

Among the components of vegetable, fruits, and tea, quercetin is suggested to play a pivotal role in the prevention of lifestyle‐related diseases. Some epidemiological studies have indicated that quercetin reduces the risk of cardiovascular diseases 1, 2. The mean estimated daily quercetin intake by residents of a town in Japan was 16 mg/day in summer and 18 mg/day in winter 3. Measurable amount of quercetin was taken from onions, tea, and other vegetables throughout the year. Supplementation with quercetin alleviated streptozotocin‐induced diabetic symptoms, and a Western‐style diet‐induced hepatic fat accumulation in mice 4, 5. Reduction of oxidative stress was thought to be an important mechanism for alleviating diabetic symptoms and liver steatosis in different mouse models 4, 5.

Obesity induces the activation and accumulation of proinflammatory cells in visceral adipose tissue (VAT), and this chronic inflammation may alter VAT function, leading to systemic insulin resistance related to metabolic syndrome 6, 7, 8. VAT is an important target organ for preventing metabolic syndrome. The inflammatory cytokine tumor necrosis factor α (TNFα), which is produced by macrophages, monocytes, and adipocytes, induces insulin resistance, whereas the adipocytokine adiponectin decreases insulin resistance 9, 10. Supplementation with 0.05% quercetin significantly suppressed visceral fat accumulation and elevation of plasma insulin and TNFα levels in mice fed a high‐fat, high‐sucrose, and high‐cholesterol Western diet for 20 weeks 5. Quercetin increased the plasma levels of adiponectin in mice fed the Western diet. Quercetin likely suppresses fat accumulation and inflammation in VAT and maintains the function in mice fed the Western diet. To determine the mechanism of the suppressive effect of quercetin on diet‐induced metabolic syndrome, we examined the effect of dietary quercetin on gene expression and function of epididymal adipose tissue (EAT) in Western diet‐induced obese mice.

## 2 Materials and methods

### 2.1 Animals and treatments

Five‐week‐old male C57BL/6J mice were obtained from the Institute for Animal Reproduction, Charles River Japan Inc. (Ibaraki, Japan). The mice were housed at 24 ± 1°C, 55 ± 5% humidity, and 12 h light/dark photocycles (dark period from 8:00 to 20:00), with free access to water and a standard nonpurified diet (NMF, Oriental Yeast Co., Tokyo, Japan) for 1 week prior to the experiment. The animals were treated in accordance with the basic guidelines of the Ministry of Agriculture, Forestry, and Fisheries for laboratory animal studies, which were approved by the review board for animal ethics at our institute (permission numbers: H24‐035, H24‐048, and H25‐039).

The mice were divided into three groups, housed in groups of three per cage, and fed one of the following diets: AIN93G (control); a high‐fat, high‐cholesterol, and high‐sucrose Western‐style diet [39.9% of energy from fat (20% unsalted butter, 1% soy oil, 0.15% cholesterol) and 34.0% of energy from sucrose (Oriental Yeast Co.); or the same Western diet supplemented with 0.05% quercetin for 18 weeks. Thereafter, the animals were killed by bleeding under anesthesia with pentobarbital (100 mg/kg), and blood, adipose tissues, and other organs were immediately collected.

### 2.2 Measurements of blood constituents and oxidative stress markers

Blood glucose levels were measured using a glucose test meter (ARKRAY Inc., Kyoto, Japan). Plasma total cholesterol and 8‐isoprostane were measured using commercial kits (Wako Pure Chemicals Industries, Osaka, Japan and Cayman Chemical Company, MI, USA, respectively). Plasma insulin, lectin‐like oxidized low‐density lipoprotein receptor‐1 (Lox‐1), IFNγ, and other adipokine concentrations were measured using commercial ELISA kits (Sibayagi, Gunma, Japan, eBioscience, San Diego, CA, USA, ALPCO Diagnostics, Salem, NH, USA or R&D systems Minneapolis, MN, USA) according to the manufacturer's instructions. Lipid peroxidation in EAT was measured as malondialdehyde (MDA) levels using a TBARS Assay Kit (Cayman Chemical Company).

### 2.3 RNA isolation, cDNA microarray analysis, and mitochondrial DNA isolation

Total RNA was extracted from EAT using TRIzol Reagent (Life Technologies Corporation, CA, USA) and an RNeasy Midi Kit (Qiagen KK, Tokyo, Japan) according to the manufacturer's instructions. Fragmented biotin‐labeled cRNA was synthesized from the total RNA of each mouse using the GeneChip 3′ IVT Express Kit (Affymetrix Japan KK, Tokyo, Japan), and then hybridized to a GeneChip Mouse Genome 430 2.0 array (Affymetrix Japan KK). After hybridization, the probe array was washed and stained using GeneChip Fluidics Station 450 (Affymetrix), and then scanned using the GeneChip Operation Software ver. 1.4 (GeneChip Scanner 3000; Affymetrix Japan KK). The data have been deposited in NCBI's gene expression omnibus (GEO) 11, and can be accessed through GEO Series accession number GSE71367.

Analysis of DNA microarray data was performed using Microarray Suite 5.0 (MAS5; Affymetrix) and GeneSpring ver. 11.5 (Agilent Technologies, CA, USA). Statistical analysis of the differences in gene expression levels among the three groups of mice was performed using Welch's one‐way ANOVA, followed by Tukey's post hoc test with Benjamini–Hochberg multiple corrections.

Ingenuity Pathway Analysis (Ingenuity Systems, CA, USA) is a commercial web‐based software package that enables analysis of data from gene expression. This analysis identifies biological functions that are most significant to the dataset and upstream regulators predicted to be activated or inhibited by treatment. Therefore, we analyzed genes that were significantly upregulated or downregulated between two groups of mice using Ingenuity Pathway Analysis. A right‐tailed Fisher's exact test was used to calculate a *p* value denoting the probability that each biological function for that dataset was due to its change alone. An activation *z*‐score was calculated as a measure of the activation of biological function and functional or translational activation of upstream regulators. An absolute *z*‐score below (inhibited) or above (activated) 2 was considered to be significant.

For mitochondrial (mt) DNA analysis, total DNA was isolated from EAT using TRIzol Reagent according to the manufacturer's instructions. The concentration and purity of the isolated DNA were determined using GeneQuant 100 (GE Healthcare Japan, Tokyo, Japan).

### 2.4 Quantitative RT‐PCR and Western blotting

Quantitative RT‐PCR was performed with an ABI PRISM 7000 Sequence Detection System (Applied Biosystems, CA, USA) using SYBR Green PCR Master Mix (Applied Biosystems) according to the manufacturer's protocol. The sequences of primers used for quantitative RT‐PCR are shown (Supporting Information Table 1). The relative amount of each transcript was normalized to the amount of *Gapdh* transcript in the same cDNA.

mtDNA was amplified using primers specific for the mitochondrial cytochrome b (CytB) gene and normalized to genomic DNA by amplification of the large ribosomal protein p0 (36B4) nuclear gene. The primers are shown (Supporting Information Table 1), and data are expressed as mitochondrial genomes per diploid nuclei.

Western blotting was performed as previously described 12. The antibodies were purchased from Santa Cruz Biotechnology (Dallas, TX, USA), and the protein bands on the X‐ray film (Amersham Hyperfilm ECL; GE Healthcare) were quantified using ImageJ software (National Institutes of Health, MD, USA).

### 2.5 Immunohistochemistry

EAT was fixed in phosphate buffered 4% paraformaldehyde and embedded in paraffin. For immunohistochemistry, deparaffinized sections were rehydrated, and endogenous peroxide was inactivated by 3% H_2_O_2_. To reduce nonspecific staining, the sections were incubated in Block Ace (DS Pharma Biomedical Co., Osaka, Japan). After incubation with anti‐mouse Mac2 antibody (Cedarlane Laboratories, Ontario, Canada), sections were treated with biotinylated rabbit anti‐rat secondary antibody (Vector Laboratories, CA, USA) and then avidin–biotin complex (Vector Laboratories). The reaction products were visualized using diaminobenzidine and counterstained with hematoxylin.

### 2.6 Stromal vascular cell preparation and flow cytometry

Small pieces of EAT were agitated in Krebs–Ringer bicarbonate buffer supplemented with heparin. The floating tissues were collected and treated with collagenase type 1 (Wako Pure Chemicals Industries) in buffer supplemented with BSA. Stromal vascular cells were filtered, washed, incubated either with T lymphocyte subset antibody cocktail (PE‐Cy7 anti‐mouse CD3e, PE anti‐mouse CD4, and FITC anti‐mouse CD8) or the isotype control (Becton Dickinson and Company, NJ, USA), and then analyzed by flow cytometry with BD FACSCanto II and BD FACSDiva software (Becton Dickinson and Company).

### 2.7 Measurement of quercetin and the metabolites in the tissues

EAT was homogenized with 0.1% phosphate buffer (pH 5.3). Quercetin and the metabolites were extracted with acetonitrile or ethyl acetate after treatment with sulfatase possessed β‐glucuronidase activity (Sigma–Aldrich, MO, USA), respectively, and then quantified as quercetin and isorhamnetin by the ACQUITY UPLC system connected to a XEVO TQD equipped with a Zspray ion source (Waters, MA, USA). Data acquisition and mass spectrometric evaluation were conducted using MassLynx software (ver. 4.1, Waters). The HPLC conditions were as follows: the ratio of acetonitrile in 0.1% formic acid was linearly increased from 10 to 50% over 15 min at 0.2 mL/min on a Zorbax300 SB‐C8 column (2.1 × 150 mm, 5 μm; Agilent Technologies) at 40°C. Capillary voltage (4 kV), source temperature (150°C), desolvation temperature (500°C), cone gas flow (50 L/h), and desolvation gas flow (800 L/h) were optimized for selected reaction monitoring (SRM) intensity. Cone voltage, collision energy, and the SRM transitions were 64 V, 30 V, and *m/z* 303/153 for quercetin; 56 V, 34 V, and *m/z* 317/153 for isorhamnetin; 32 V, 14 V, and *m/z* 479/303 for quercetin 3‐o’‐glucronide; 42 V, 22 V, and *m/z* 383/303 for quercetin 3‐o‐sulfate; and 66 V, 32 V, and *m/z* 275/154 for the internal standard, genistein‐*d*_4_, respectively.

### 2.9 Statistical analysis

Statistical analyses were performed using Excel 2013 (Microsoft, Tokyo, Japan) and R‐3.2.2 (R Foundation for Statistical Computing, Vienna, Austria). Data were expressed as the arithmetic mean ± SEM. The significance of differences between two and three groups were determined by Student's *t*‐test and ANOVA, followed by two‐tailed multiple *t*‐tests with Holm correction. A *p* value of <0.05 was considered statistically significant.

## 3 Results

### 3.1 Effect of quercetin on Western diet‐induced obesity and blood constituents in mice

**Figure 1:**
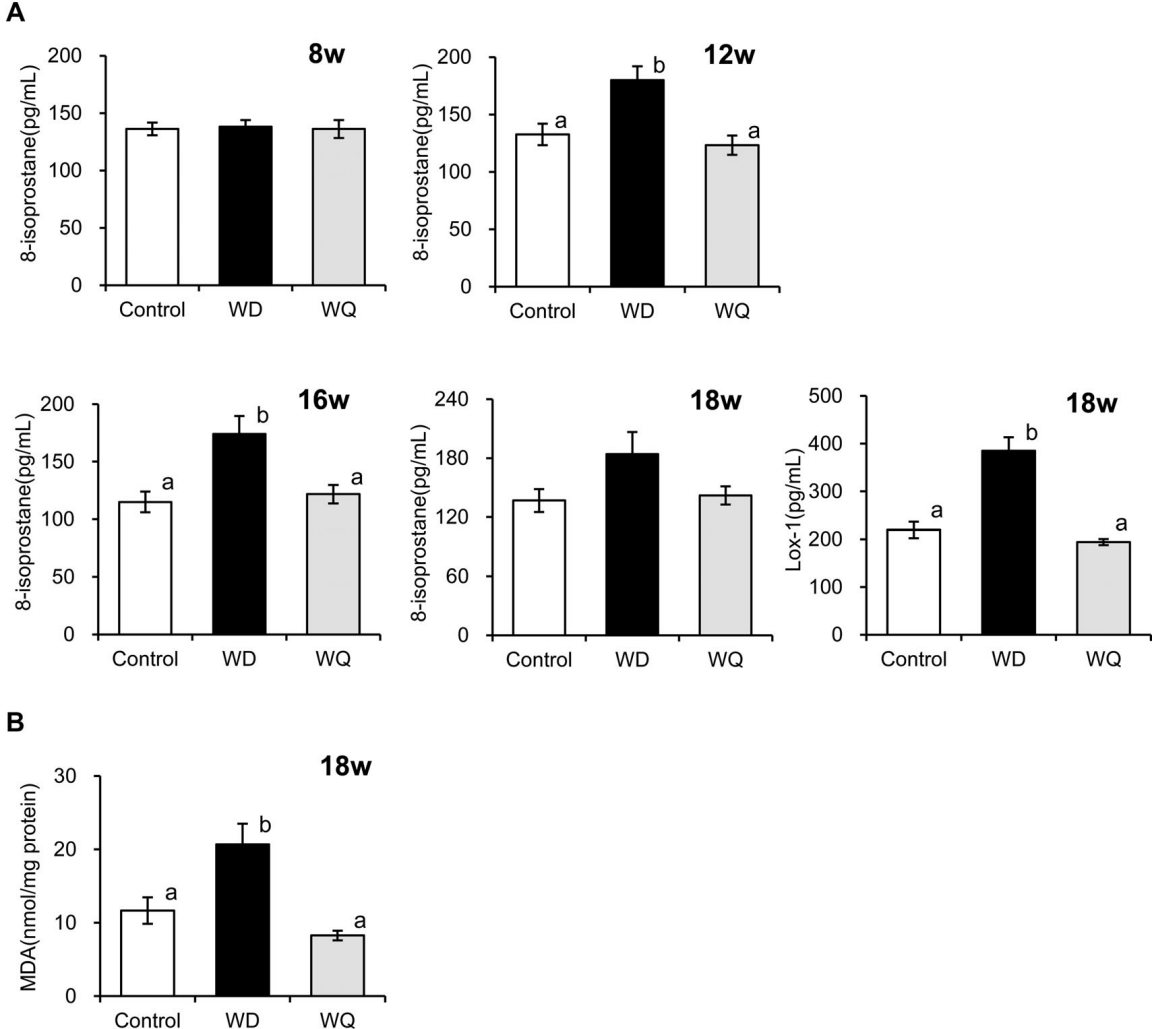
Reduction of oxidative stress marker levels in plasma (A) and epididymal adipose tissue (B) by quercetin in mice fed a Western diet. C57BL/6J mice were fed a control diet (Control), Western diet (WD), or Western diet containing 0.05% quercetin (WQ). Values are expressed as the means ± SEM of 7–9 mice in each group. Different superscripts indicate significant differences (*p* < 0.05, two‐sided).

**Figure 2:**
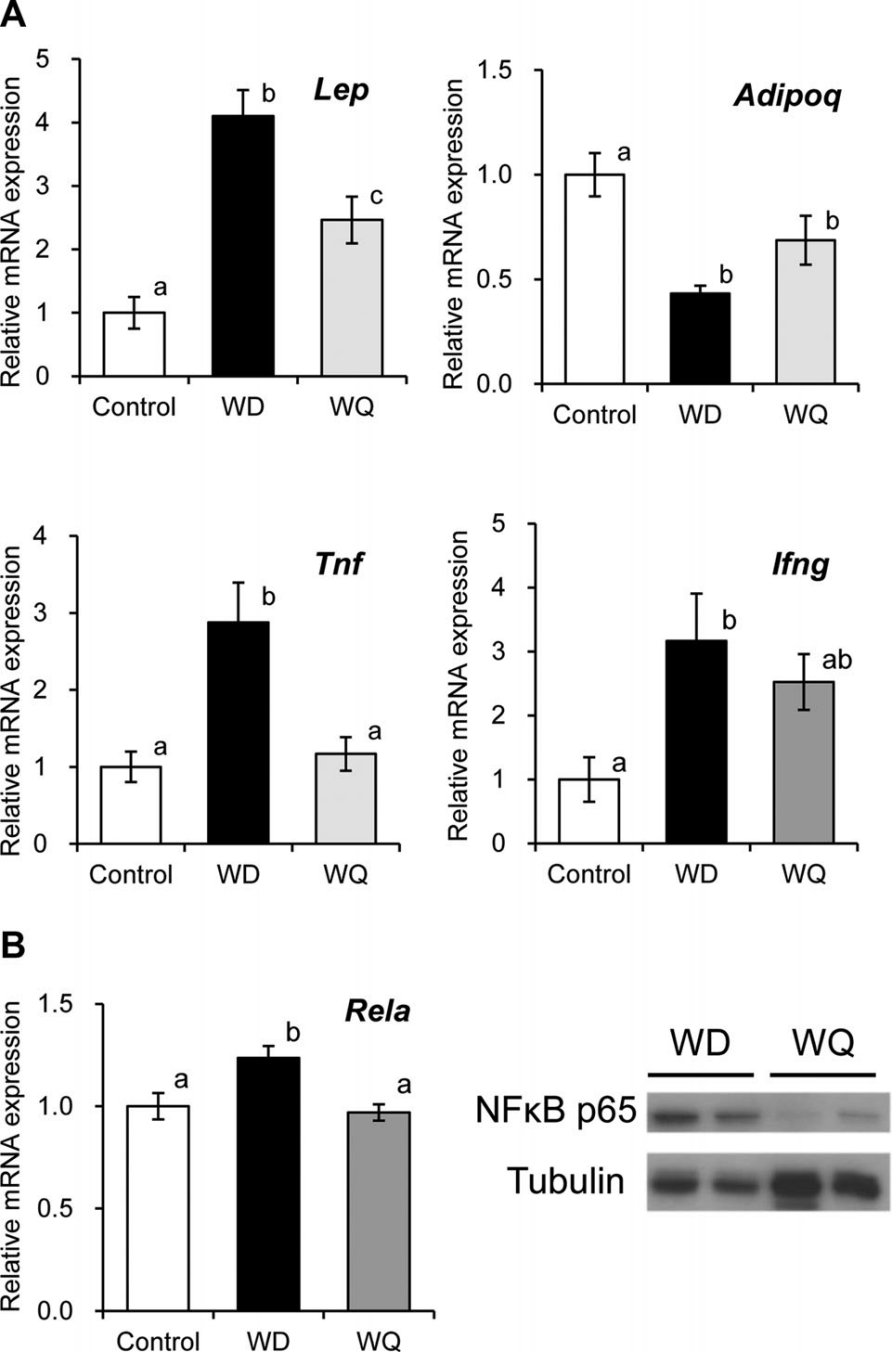
Effect of quercetin on Western diet‐altered gene expression of adipocytokines, proinflammatory cytokines, and the proinflammatory transcription factor NFκB in the epididymal adipose tissue. Mice were fed a control diet (Control), Western diet (WD), or Western diet containing 0.05% quercetin (WQ) for 18 weeks. Quercetin suppressed the gene expression of leptin (*Lep*) and TNFα (*Tnf*) (A) and the gene (*Rela*) and protein expression of NFκB p65 (B) in mice fed a Western diet. Values are expressed as the means ± SEM of nine mice in each group. Different superscripts indicate significant differences (*p* < 0.05, two‐sided).

Supplementation with quercetin significantly reduced body weight, and the levels of blood glucose, plasma insulin, cholesterol, leptin, and TNFα in mice fed the Western diet for 18 weeks (Table 1). Dietary quercetin suppressed the increase in the oxidative stress markers 8‐isoprostane and Lox‐1 in the plasma of mice fed the Western diet for 12, 16, or 18 weeks (Fig. 1A). Induction of the lipid peroxidation marker MDA was suppressed by quercetin in the EAT of mice fed the Western diet for 18 weeks (Fig. 1B). Further, quercetin strongly suppressed the increase in the expression of TNFα and the adipocytokine leptin in the Western diet‐induced obese mice EAT (Fig. 2A). TNFα induces the expression of proinflammatory cytokines/adipocytokines, including TNFα, by activating the oxidative stress‐sensitive transcription factor NFκB 13, 14, 15. Relative NFκB p65 protein levels in the EAT of mice fed the Western diet containing quercetin was 0.5 ± 0.1‐fold that of mice fed the Western diet. Quercetin reduced the gene and protein expression of NFκB p65 in the mice fed the Western diet (Fig. 2B).

**Table 1:** Alleviation of obesity and improvement in the levels of blood constituents by quercetin in mice fed a Western diet

	Control diet	Western diet	Western diet+ 0.05% quercetin
Body weight (g)	35.66 ± 1.46^a^	45.77 ± 0.95^b^	41.69 ± 1.33^c^
Liver weight (g)	1.46 ± 0.08^a^	3.47 ± 0.28^b^	2.13 ± 0.19^c^
Kidney weight (g)	0.37 ± 0.01	0.36 ± 0.01	0.38 ± 0.01
Pancreas weight (g)	0.28 ± 0.02	0.30 ± 0.03	0.27 ± 0.02
Visceral fat (g/mouse)	2.25 ± 0.43^a^	4.34 ± 0.12^b^	3.33 ± 0.36^a,b^
Blood glucose (mg/dL)	143 ± 5^a^	224 ± 9^b^	151 ± 9^a^
Plasma insulin (ng/mL)	1.84 ± 0.47^a^	4.88 ± 0.67^b^	2.22 ± 0.32^a^
Plasma cholesterol (mg/dL)	76.2 ± 10.9^a^	194.0 ± 9.2^b^	123.3 ± 12.4^c^
Plasma leptin (ng/mL)	1.46 ± 0.61^a^	5.78 ± 0.58^b^	2.81 ± 0.51^a^
Plasma adiponectin (μg/mL)	4.42 ± 0.42^a^	2.87 ± 0.11^b^	3.18 ± 0.50^a,b^
Plasma resistin (ng/mL)	18.7 ± 1.2	22.2 ± 1.4	21.8 ± 1.2
Plasma TNFα (pg/mL)	13.2 ± 2.6^a^	34.9 ± 3.5^b^	19.9 ± 2.8^a^
Plasma IFNγ (pg/mL)	2.11 ± 0.33^a^	6.81 ± 1.3^b^	3.21 ± 0.58^a^

C57BL/6J mice were fed the control AIN93G diet or a Western diet containing either 0% or 0.05% quercetin for 18 weeks. Values are expressed as the mean ± SEM of 6–9 mice in each group. Different superscripts indicate significant differences (p < 0.05, two‐sided).

### 3.2 Comprehensive gene expression analysis of EAT using a DNA microarray

Comprehensive gene expression analysis using a DNA microarray showed that 4657 genes were differentially expressed in the EAT of mice fed the control diet, Western diet, and Western diet containing quercetin for 18 weeks (*n* = 9, *p* < 0.05 by one‐way ANOVA). Ingenuity Pathway Analysis showed that quercetin was predicted to decrease 104 functions that are associated with immune cells, including macrophages, lymphocytes, granulocytes, and mast cells, inflammatory responses, and free radical production, out of the 154 functions predicted to be increased by the Western diet (Table 2, Supporting Information Tables 2 and 3). Our results suggest that quercetin suppresses the accumulation, movement, and activation of macrophages, lymphocytes, and other immune cells, and the subsequent inflammation in EAT of mice fed the Western diet (Table 2).

**Table 2:** Predicted biological functions decreased by quercetin in EAT of Western diet‐induced obese mice

Predicted biological function decreased by quercetin	*p*‐value	Activation z‐score
Cell death, cell viability, binding, phagocytosis, activation, proliferation, development, engulfment, quantity of **blood cells**	2.01 × 10^–7^ ‒ 2.57 × 10^–2^	–4.055 to –2.000
Cell death, adhesion, proliferation of **immune cells**	2.88 × 10^–7^ ‒ 8.57 × 10^–3^	–3.385 to –2.717
Cell death, cell viability, phagocytosis, recruitment, activation, adhesion, engulfment, cell movement, homing, migration, chemotaxis, accumulation of **myeloid cells**	3.97 × 10^–7^ ‒ 3.26 × 10^–2^	–4.515 to –2.069
Cell viability, cytotoxicity, polarization, phagocytosis, recruitment, activation, binding, immune response, development, cell movement, homing, migration, transmigration, quantity of **leukocytes**	4.34 × 10^–8^ ‒ 3.40 × 10^–2^	–5.755 to –2.056
Cell viability, activation, proliferation, cell movement, homing, migration, transmigration, quantity of **mononuclear leukocytes**	2.18 × 10^–3^ ‒ 2.11 × 10^–2^	–3.444 to –2.056
Cell death, response, recruitment, engulfment, cell movement, migration, accumulation of **antigen presenting cells**	1.59 × 10^–5^ ‒ 2.74 × 10^–3^	–3.770 to –2.021
Cell death, respiratory burst, recruitment, cell movement, chemotaxis, migration, accumulation of **macrophages**	5.51 × 10^–6^ ‒ 2.13 × 10^–2^	–2.828 to –2.000
Cell death of **bone marrow‐derived macrophages**	1.08 × 10^–2^	–2.570
Cell death, recruitment, activation, response, engulfment, localization, homing, cell movement, chemotaxis, migration, accumulation of **phagocytes**	2.59 × 10^–7^ ‒ 8.97 × 10^–3^	–4.820 to –2.000
Cell movement, migration of **dendritic cells**	1.12 × 10^–2^ ‒ 1.84× 10^–2^	–2.784 to –2.468
Cytotoxicity, cell viability, polarization, activation, proliferation, development, cell movement, homing, chemotaxis, quantity, homeostasis, migration of **lymphocytes**	1.13 × 10^–3^ ‒ 3.15 × 10^–2^	–3.155 to –2.014
Cell movement, homing, transmigration, development, homeostasis, migration, quantity of **T lymphocytes**	8.55 × 10^–4^ ‒ 2.71 × 10^–2^	–2.567 to –2.177
Cell viability of **B lymphocytes**	1.12 × 10^–2^	–2.195
Cytotoxicity of **natural killer cells**	1.43 × 10^–3^	–2.986
Cell viability, activation of **mast cells**	8.09 × 10^–4^ ‒ 3.02 × 10^–2^	–2.449 to –2.138
Binding, adhesion, cell movement, homing, chemotaxis, accumulation of **granulocytes**	1.43 × 10^–6^ ‒ 3.71 × 10^–2^	–3.646 to –2.011
Response of **eosinophils**	7.05 × 10^–3^	–2.236
Binding, recruitment, cell movement, migration, accumulation of **neutrophils**	2.93 × 10^–7^ ‒ 3.10 × 10^–2^	–2.979 to –2.138
Generation, production, synthesis of **reactive oxygen species**	5.09 × 10^–9^ ‒5.21 × 10^–9^	–3.462 to –2.210
Generation, production of **superoxide**	3.13 × 10^–4^ ‒ 1.79 × 10^–3^	–2.779 to –2.200

*p* values < 0.05 were considered to be a significant dataset of the targets of biological function (Fisher's exact test). An absolute *z*‐score below (inhibited) or above (activated) 2 was considered significant.

IFNγ, which induces adipose inflammation and insulin resistance and some other molecules were predicted to be upstream regulators that are inhibited or activated by quercetin in the EAT of mice fed the Western diet (Table 3). Among them, IFNγ; microRNA‐223, which suppresses proinflammatory pathways and enhances the alternative anti‐inflammatory response in obese adipose tissues; cholesterol; and transcription factor Spi‐B, which is specifically expressed in lymphoid cells, were predicted to be activated by the Western diet (Table 3 and Supporting Information Table 4 (1)) 16, 17. Table 4 shows the top five canonical pathways of genes that were significantly altered by quercetin in the tissues of mice fed the Western diet. Other than the pathway associated with immune responses, quercetin markedly increased most of the gene expression associated with “mitochondrial dysfunction” and “oxidative phosphorylation.” This result suggests that quercetin increased mitochondrial oxidative phosphorylation in the Western diet‐induced obese mice.

**Table 3:** Upstream regulators predicted to be activated or inhibited by quercetinin EAT in mice fed a Western diet

Prediction	
Activated upstream regulator	Inhibited upstream regulator
LEPR (16 target molecules in dataset), NPC1 (5)	IFNγ (57 target molecules in dataset), mir‐223 (18), ABCB6 (7), cholesterol (7), ALOX5 (4) SPI1 (9), SPIB (11)

*p* values < 0.05 were considered to be a significant dataset of the targets of each upstream regulator (Fisher's exact test). An absolute *z*‐score below (inhibited) or above (activated) 2 was considered significant.

LEPR, leptin receptor; NPC1, NiemannPick disease, type C; mir‐223, microRNA‐223; ABCB6, ATP‐binding cassette, sub‐family B (MDR/TAP), member 6; ALOX5, arachidonate 5‐lipoxygenase; SPI1, transcription factor PU.1; SPIB, Spi‐B transcription factor (Spi‐1/PU.1 related).

**Table 4:** Top 5 canonical pathways of genes that were significantly altered by quercetinin EAT in mice fed a Western diet

Ingenuity canonical pathways	*p*‐value	Genes altered by quercetin/ genes in the canonical pathway	Up regulated genes/ downregulated genes
**Mitochondrial dysfunction**	3.49 × 10^–10^	40/136 (0.294)	36/4
Fcγ receptor‐mediated phagocytosis in macrophages and monocytes	7.11 × 10^–9^	29/89 (0.326)	4/25
Complement system	7.03 × 10^–8^	14/27 (0.519)	0/14
**Oxidative phosphorylation**	3.28 × 10^–7^	25/82 (0.305)	25/0
CD28 signaling in T‐helper cells	5.51 × 10^–6^	27/106 (0.255)	4/23

The functions and canonical pathways that were most significant to the dataset were identified by Ingenuity Pathway Analysis.

### 3.3 Quercetin suppressed the accumulation and activation of immune cells in the Western diet‐induced obese mice EAT

**Figure 3:**
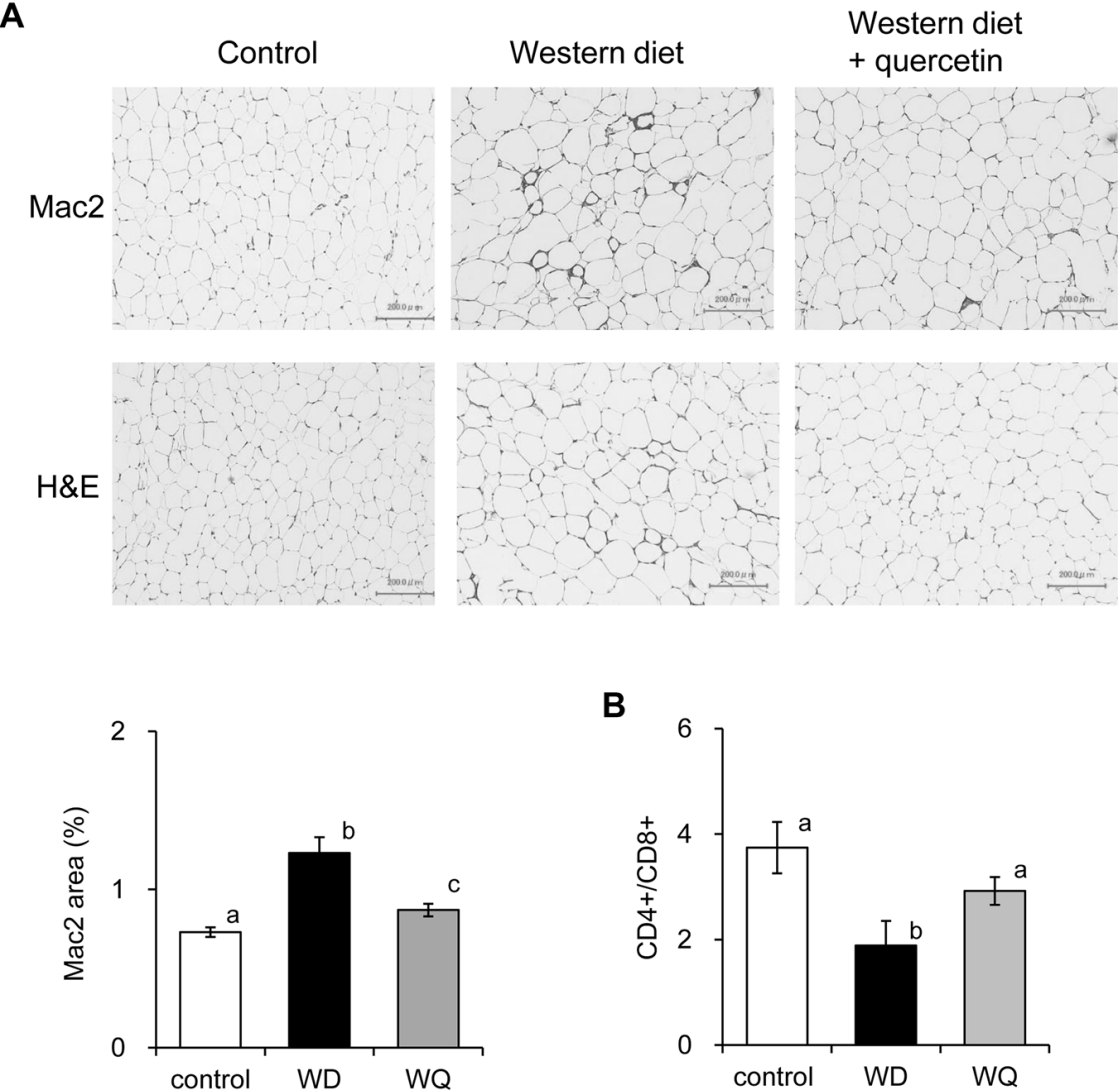
Quercetin suppressed the accumulation of macrophages and increase in the ratio of CD8^+^ T cells in stromal vascular cells in the epididymal adipose tissue (EAT) of mice fed a Western diet. (A) Representative photomicrographs showing EAT stained with anti‐Mac2 antibody (Mac2) or hematoxylin and eosin (H&E) and the percentage of Mac2‐stained area. (B) The CD4^+^/CD8^+^ lymphocyte ratios as determined by a flow cytometer in the stromal vascular cells of EAT. Mice were fed a control diet (Control), Western diet (WD), or Western diet containing 0.05% quercetin (WQ) for 18 weeks. Values are expressed as the means ± SEM of five or six mice in each group. Different superscripts indicate significant differences (*p* < 0.05, two‐sided).

**Figure 4:**
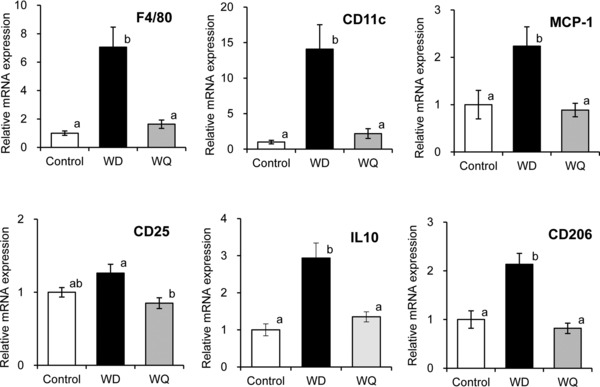
Quercetin suppressed the Western diet‐induced expression of genes associated with immune cells and the responses in epididymal adipose tissue. Mice were fed a control diet (Control), Western diet (WD), or Western diet containing 0.05% quercetin (WQ) for 18 weeks. Values are expressed as the means ± SEM of nine mice in each group. Different superscripts indicate significant differences (*p* < 0.05, two‐sided).

Immunohistochemical analysis of Mac2 positive macrophages showed that the Western diet‐induced accumulation of macrophages was suppressed by quercetin in EAT (Fig. 3A). mRNA expression of the macrophage marker F4/80 and the inflammation‐related expression of CD11c and monocyte chemotactic protein‐1 were increased by the Western diet and decreased by quercetin to the control levels (Fig. 4). However, supplementation with quercetin significantly reduced the expression of CD206, a marker of the anti‐inflammatory M2 macrophages in EAT of mice fed the Western diet (Fig. 4).

The ratio of CD8^+^ to CD4^+^ T cells increased in obese adipose tissues, contributing to macrophage recruitment in mice 7. Our results show that quercetin significantly increased the ratio of CD4^+^ to CD8^+^ T cells in the tissues of the Western diet‐induced obese mice (Fig. 3B). Supplementation with quercetin reduced the expression of the T‐cell activation marker CD25 expressed on regulatory T cells, and the anti‐inflammatory cytokine IL10 in the Western diet‐induced obese mice (Fig. 4). Thus, dietary quercetin suppressed the accumulation of both inflammatory and anti‐inflammatory macrophages, T cells, and cytokines in the EAT of the Western diet‐induced obese mice.

### 3.4 Quercetin suppressed the expression of NADPH oxidase and increased the expression of antioxidant enzymes in EAT

**Figure 5:**
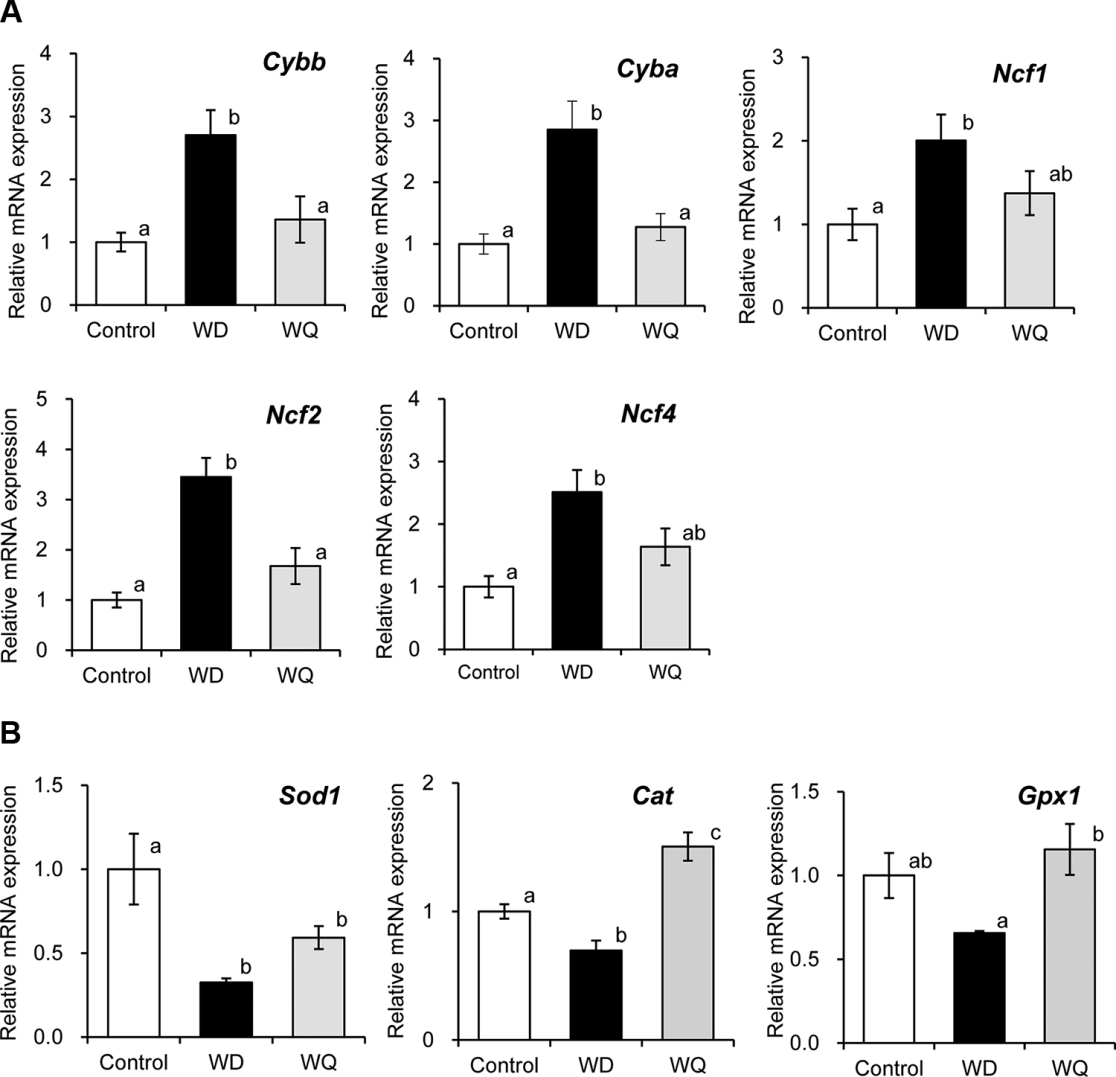
Quercetin suppressed the expression of NADPH oxidase subunits (A) and increased the expression of antioxidant enzymes (B) in the epididymal adipose tissue of mice fed a Western diet. Mice were fed a control diet (Control), Western diet (WD), or Western diet containing 0.05% quercetin (WQ) for 18 weeks. Values are expressed as the means ± SEM of nine mice in each group. Different superscripts indicate significant differences (*p* < 0.05, two‐sided).

DNA microarray analysis suggested that quercetin reduced the generation of reactive oxygen species in EAT of mice fed the Western diet (Table 2). To elucidate the regulation of antioxidant status by quercetin, we then determined the expression of the NADPH oxidase subunits and the antioxidant enzymes by quantitative RT‐PCR analysis. We found that quercetin significantly suppressed the induction of NADPH oxidase expression and increased the expression of the antioxidant enzymes superoxide dismutase 1 (*Sod1*), glutathione peroxidase 1 (*Gpx1*), and catalase (*Cat*) (Fig. 5). *Sod1*, *Gpx1*, and *Cat* are known to be regulated by nuclear factor (erythroid derived 2)‐like 2 (Nrf2) in EAT of mice fed the Western diet (Fig. 5). However, expression of Nrf2 (*Nfe2l2*) and the Nrf2‐responsive genes oxygenase 1 (HO‐1, *Hmox1*) and oxidoreductase 1 (*Nqo1*) were not significantly increased by quercetin in the tissues of mice fed the Western diet (data not shown).

### 3.5 Quercetin increased mitochondrial DNA content in EAT

**Figure 6:**
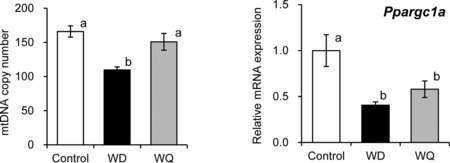
Quercetin increased mitochondrial DNA content in the epididymal adipose tissue of mice fed a Western diet. Mice were fed a control diet (Control), Western diet (WD), or Western diet containing 0.05% quercetin (WQ) for 18 weeks. Values are expressed as the means ± SEM of six or nine mice in each group. Different superscripts indicate significant differences (*p* < 0.05, two‐sided).

We then determined the mtDNA copy number in EAT, and quercetin significantly increased the mtDNA copy number in the tissues of mice fed the Western diet (Fig. 6A). Although the Western diet suppressed the expression of peroxisome proliferative‐activated receptor gamma, coactivator 1 alpha (PGC‐1α, *Ppargc1a*), a master transcription regulator of oxidative phosphorylation and mitochondrial biogenesis, quercetin did not significantly increase the expression of *Ppargc1a* in the tissues of mice fed the Western diet (Fig. 6B).

### 3.6 Nonfasting levels of quercetin and its metabolites in the EAT of mice fed a Western diet containing quercetin for 18 weeks

**Figure 7:**
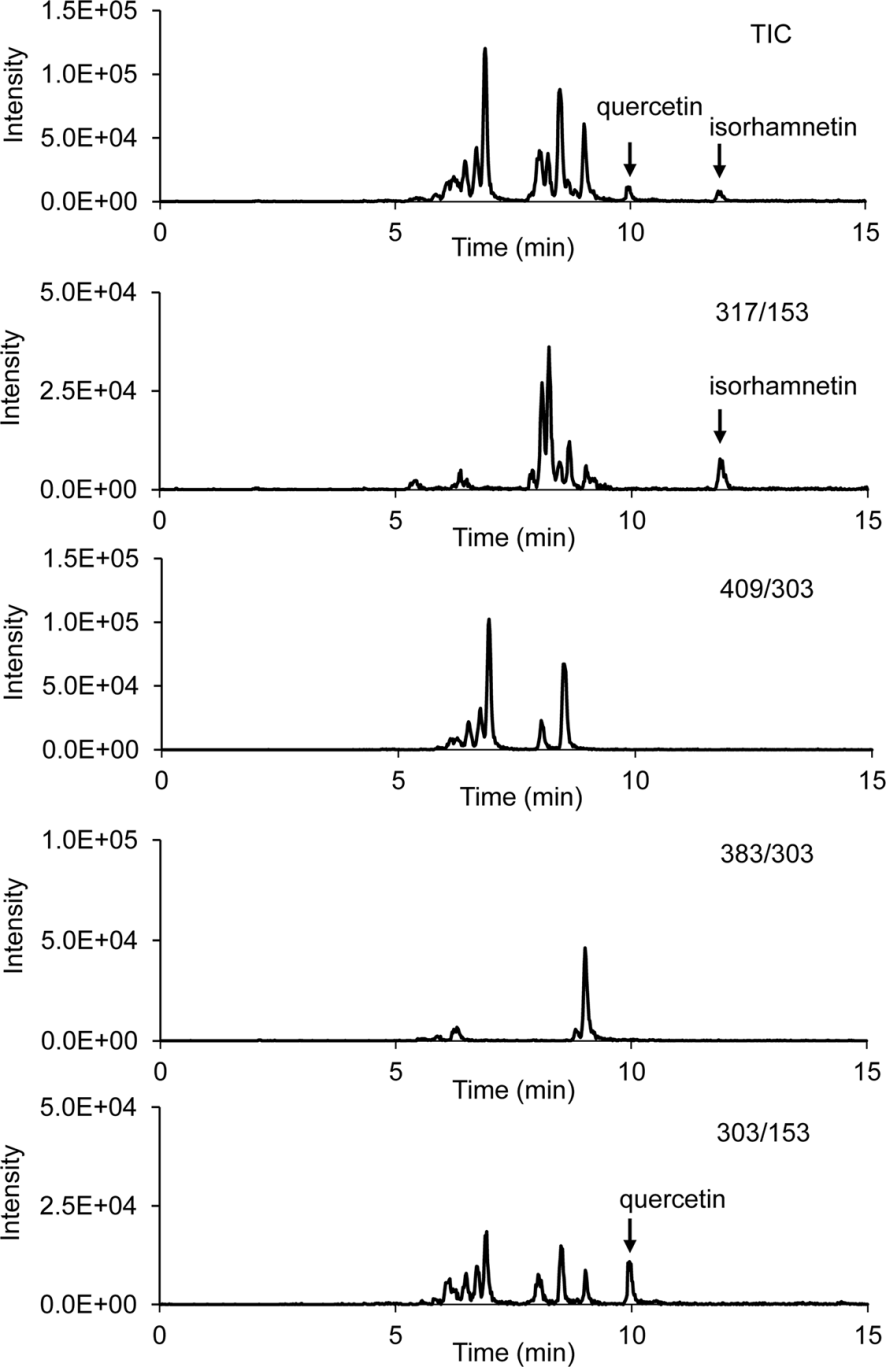
Representative selected reaction monitoring chromatogram of quercetin metabolites in the epididymal adipose tissue of mice fed a Western diet containing quercetin for 18 weeks. Mice were fed a Western diet containing 0.05% quercetin (WQ) for 18 weeks. Selected reaction monitoring (SRM) transitions for detecting methylated, glucuronidated, and sulfated quercetin in were *m/z* 317/153, *m/z* 479/303, and *m/z* 383/303, respectively. SRM transition for detecting quercetin was *m/z* 303/153.

After 2 hour fasting, mice were killed and the EATs were immediately removed, washed with PBS, and frozen using liquid nitrogen until the measurement of quercetin and the metabolites. LC‐MS/MS analysis showed that methylated, glucuronidated, and/or sulfated quercetin metabolites are present in the EAT of mice fed the Western diet containing quercetin for 18 weeks (Fig. 7). The levels of the quercetin and isorhamnetin aglycones were 5.1 ± 0.8 pmol/g and 1 ± 0.3 pmol/g in EAT of mice fed the Western diet containing quercetin, respectively. Quercetin metabolites were hydrolyzed through sulfatase and β‐glucuronidase activity and quantified as quercetin and isorhamnetin, respectively. The total quercetin and isorhamnetin levels were 186.6 ± 25.6 pmol/g and 74.9 ± 20.7 pmol/g in EAT. Although the concentrations of quercetin and its metabolites may be affected by the intake of diet within several hours, the mice probably maintained similar levels of quercetin and metabolites in the EAT after the chronic intake of quercetin. The concentrations of the quercetin and isorhamnetin aglycones were much lower than those of the total conjugated quercetin and isorhamnetin.

## 4 Discussion

Comprehensive gene expression analysis was used to reveal the characteristics of the effect of dietary quercetin on the EAT of mice fed the Western diet. Supplementation with quercetin suppressed the increase and activation of immune cells in the tissues of mice fed the Western diet. Quercetin markedly increased gene expression associated with mitochondrial oxidative phosphorylation, likely due to suppressed reduction of the mtDNA copy number in the EAT of the diet‐induced obese mice.

Adipocyte class II major histocompatibility complex (MHCII), required for antigen presentation and induces antigen‐specific CD4^+^ T‐cell activation, increased in mice early on during the high‐fat diet challenge, paralleling increases in, and the activation of proinflammatory CD4^+^ Th1 cells, and preceding macrophage accumulation and proinflammatory M1 polarization 18. Early increases in adipocytokine leptin may promote T‐cell proliferation and secretion of IFNγ, which promotes adipocyte MHCII expression 18. IFNγ promotes adipocyte lipolysis, macrophage infiltration, and M1 polarization 18, 19. IFNγ and TNFα produced by M1 macrophages and adipocytes induce systemic inflammation and insulin resistance 9, 14, 19, 20. CD8^+^ and CD4^+^ T cells increase in the VAT of diet‐induced obese mice and promote the recruitment and activation of macrophages 21. High‐fat diets increase the number and activation of resident NK cells, and produce IFNγ in VAT 8. B cells, which are recruited early on in VAT in response to a high‐fat diet, induce MHC‐dependent proinflammatory cytokine production by both CD4^+^ and CD8^+^ T cells and produce pathogenic proinflammatory IgG2c antibody 22. Dendritic and mast cells are increased in obese white adipose tissues, and contribute to promote macrophage infiltration of adipose tissues and glucose intolerance, respectively 23, 24. Moreover, high‐fat diets increase neutrophil recruitment to the adipose tissue and facilitate macrophage infiltration by producing chemokines and cytokines 25.

Here, the gene expression associated with the proliferation, migration, recruitment, and activation of immune cells was induced by the Western diet in the EAT of mice (Supporting Information Table 2). Quercetin reduced the gene expression associated with MHCII antigen presentation, antigen‐specific T‐cell activation (“CD28 signaling in T‐helper cells” in Table 2 (2)), and accumulation of macrophages. Our results suggest that quercetin reduces the NFκB‐mediated induction of TNFα and other proinflammatory cytokines/adipokines. Although quercetin did not significantly reduce the elevations of plasma IFNγ levels and adipose IFNγ expression, comprehensive gene expression analysis predicted that quercetin inhibits IFNγ as an upstream regulator. Quercetin probably suppresses the induction of leptin and IFNγ expression in EAT and the subsequent antigen‐specific CD4^+^ T‐cell activation and macrophage infiltration and activation.

Leptin receptor, which is inversely related to the increased expression of leptin in mice fed a high‐fat diet, and Niemann–Pick disease, type C, which mediates intracellular cholesterol trafficking, were predicted to be upstream regulators activated by quercetin 26, 27. Other than IFNγ, the proinflammatory arachidonate 5‐lipoxygenase and Spi‐1 proto‐oncogene (Spi1), which are highly expressed in B cells, mast cells, macrophages, and neutrophils, were predicted to be upstream regulators inhibited by quercetin 17, 28. Quercetin was predicted to decrease functions associated with the viability, activation, or migration of B cells, NK cells, mast cells, dendritic cells, and neutrophils. The expression of CD11c, which is a marker of dendritic cells and M1 macrophages, was decreased by quercetin in the tissues of mice fed the Western diet. The complement system, which is included in the process of promoting adipocyte inflammation and insulin resistance, may be suppressed by quercetin 29.

High‐fat diets induce the expression of IL10, an anti‐inflammatory cytokine, and increase the number of alternative anti‐inflammatory M2 macrophages in EAT, although the increased ratio of M1‐to‐M2 macrophages ultimately induces insulin resistance 30. RT‐PCR showed that quercetin suppresses the induction of the expression of IL10 and the M2 macrophage marker CD206 in mice fed the Western diet. Thus, quercetin suppresses the accumulation and activation of immune cells and the subsequent inflammation and systemic insulin resistance, probably by suppressing the expression of leptin, TNFα, and possibly IFNγ. Suppression of the activation of NFκB is likely to reduce the production of TNFα and other proinflammatory cytokines.

The main effect of dietary quercetin on organs is the reduction of oxidative stress marker levels 4, 5, 12. As Kawai et al. and Santos et al. reported, methylated, glucuronidated, and/or sulfated quercetin metabolites found in the plasma of mice fed the Western diet containing quercetin for 18 weeks (Supporting Information Fig. 1) 31, 32. They showed that the plasma quercetin metabolites retained the antioxidative activity; however, continuous feeding of the diet containing quercetin increased the levels of methylated metabolites and thereby reduced the antioxidative activity 31, 32. In our study, quercetin suppressed the increase in the oxidative stress markers 8‐isoprostane or Lox‐1 in the plasma of mice fed the Western diet for 12–18 weeks. Although the plasma 8‐isoprostane levels in mice fed the Western diet containing quercetin were not significantly lower than that of the mice fed the Western diet after 18 weeks, the levels in mice fed the Western diet containing quercetin were comparable between 8 and 18 weeks of feeding. We also identified the methylated, glucuronidated, and/or sulfated quercetin metabolites in the EAT of mice fed the Western diet containing quercetin. Dietary quercetin significantly suppressed the increase in the levels of the lipid peroxidation marker MDA and gene expression associated with the production of reactive oxygen species in the EAT of mice fed the Western diet. Although quercetin may increase the expression of antioxidant enzymes by activating the Nrf2 pathway in the liver of normal control mice, supplementation of quercetin did not significantly increase the expression of Nrf2 or Nrf2‐regulated HO‐1 and NQO‐1 in the EAT of Western diet‐induced obese mice 12. Increased oxidative stress in white adipose tissues induces obesity, inflammation, and insulin resistance 33, 34, 35. The antioxidative effect of quercetin metabolites probably prevents the increase of reactive oxygen species and the progression of inflammation and insulin resistance by suppressing the accumulation of immune cells and the production of TNFα and other inflammatory cytokines in adipocytes in the EAT of Western diet‐induced obese mice

In this study, dietary quercetin universally suppressed Western diet‐induced gene expression associated with the accumulation and activation of immune cells, whereas it specifically increased gene expression associated with mitochondrial oxidative phosphorylation. Mitochondrial content of white adipocytes is decreased in both rodent and human obesity and correlates with insulin resistance 36, 37. A decrease in mtDNA copy number reduces β‐oxidation and impaired mitochondrial oxidative phosphorylation, which correspondingly induces insulin resistance 38, 39. The increase in the mtDNA copy number by dietary quercetin likely improves mitochondrial oxidative phosphorylation in EAT. Quercetin is an activator of adenosine monophosphate‐activated protein kinase (AMPK), which activates PGC‐1α, resulting in the upregulation of mitochondrial biosynthesis 40, 41. Previous studies showed that adipose‐specific deficiency of PGC‐1α induced insulin resistance, and activation of AMPK phosphorylation and mitochondrial biogenesis regulated insulin resistance 40, 42. Dong et al. showed that quercetin enhanced AMPKα1 phosphorylation in the EAT of mice fed a high‐fat diet 41, whereas Xu et al. reported that quercetin aglycone increased AMPK activity in 3T3‐L1 adipocytes (2 × 10^6^ cells/well) at concentrations ranging from 0.1–10 μM 43. Shen et al. showed that quercetin, and the metabolites 3′‐O‐methyl quercetin and quercetin‐3‐O‐glucuronides induce the phosphorylation of AMPK in human aortic endothelial cells 44. The adipose cell number was around 1 × 10^6^ cells/g in the EAT of 18‐week‐old mice fed a high‐fat diet 45. Quercetin metabolites may induce the gene expression associated with mitochondrial oxidative phosphorylation through the activation of AMPK and, thus, of PGC‐1α, in the EAT of mice fed the Western diet. However, here dietary quercetin did not significantly induce PGC‐1α expression or AMPK phosphorylation in the EAT of mice fed the Western diet (data not shown). Increases in reactive oxygen species induce mitochondrial dysfunction and reduce mtDNA content in white adipose tissues 37; the antioxidative effect of quercetin metabolites is likely involved in a pathway including quercetin‐increased oxidative phosphorylation‐related gene expression and mtDNA content.

In conclusion, supplementation with quercetin suppressed gene expression associated with the accumulation and activation of immune cells, including macrophages, lymphocytes, dendritic cells, and mast cells in the EAT of mice fed the Western diet for 18 weeks. Dietary quercetin suppressed the accumulation of macrophages, the decrease in the ratio of CD4^+^ to CD8^+^ T cells in EAT, and the elevation of plasma TNFα and leptin levels in Western diet‐induced obese mice. Although quercetin reduced the expression of the anti‐inflammatory M2 macrophage marker and cytokine IL10, it suppressed the progression of chronic low‐grade inflammation in adipose tissues. Quercetin increased antioxidant enzyme levels and reduced the expression of NADPH oxidase and lipid peroxidation in EAT. It also suppresses the expression of NFκB. Suppression of oxidative stress and NFκB expression likely contribute to the prevention of the accumulation and activation of immune cells and resulting chronic inflammation. Overall, quercetin increased gene expression associated with mitochondrial oxidative phosphorylation and mtDNA content.

## Supporting information

As a service to our authors and readers, this journal provides supporting information supplied by the authors. Such materials are peer reviewed and may be re‐organized for online delivery, but are not copy‐edited or typeset. Technical support issues arising from supporting information (other than missing files) should be addressed to the authors.

Supplementary Material
