# Supplementary material for: Quercetin suppresses immune cell accumulation and improves mitochondrial gene expression in adipose tissue of diet‐induced obese mice
Source: Mol Nutr Food Res. 2015 Nov 24;60(2):300–12. doi: 10.1002/mnfr.201500595 (PMC5063128; doi:10.1002/mnfr.201500595)
Supplement: Supplementary file 1 — Supplementary Material [file MNFR-60-300-s001.zip › mnfr201500595-sup-0002-Supporting Information Figure Legend.docx]

Supporting Information Figure Legend:

Figure S1. Representative selected reaction monitoring chromatogram of quercetin metabolites in the plasma of mice fed a Western diet containing quercetin for 18 weeks.

Mice were fed a Western diet containing 0.05% quercetin (WQ) for 18 weeks. Selected reaction monitoring (SRM) transitions for detecting methylated, glucuronidated, and sulfated quercetin were <i>m/z</i> 317/153, <i>m/z</i> 479/303, and <i>m/z</i> 383/303, respectively. SRM transition for detecting quercetin was <i>m/z</i> 303/153.
