# Supplementary material for: Quercetin suppresses immune cell accumulation and improves mitochondrial gene expression in adipose tissue of diet‐induced obese mice
Source: Mol Nutr Food Res. 2015 Nov 24;60(2):300–12. doi: 10.1002/mnfr.201500595 (PMC5063128; doi:10.1002/mnfr.201500595)
Supplement: Supplementary file 1 — Supplementary Material [file MNFR-60-300-s001.zip › mnfr201500595-sup-0006-Table S4.docx]

Table S4. Upstream regulators predicted to be activated or inhibited (1)^a^ and Top 5 canonical pathways of genes that were significantly altered (2)^b^ by Western diet　in epididymal adipose tissues in mice

(1)

| Prediction | |
| --- | --- |
| Activated upstream regulator | Inhibited upstream regulator |
| IL4 (104 target molecules in dataset), mir-223 (31), IFNγ (86), cholesterol (11), MYD88 (36), PPARδ (10), TNF (33), SPIB (18), IRF7 (16), IRF3 (17), TYROBP (8), | GATA6 (16 target molecules in dataset) |

^a^P values less than 0.05 were considered to be a significant data set of the targets of each upstream regulator (Fisher’s exact test). An absolute *z*-score of below (inhibited) or above (activated) 2 was considered significant.

(2)

| Ingenuity Canonical Pathways | p-value | Genes altered by quercetin  /genes in the canonical pathway | Up regulated genes /downregulated genes |
| --- | --- | --- | --- |
| Fcγ　receptor-mediated phagocytosis in macrophages and monocytes | 1.1E-08 | 40/89 (0.449) | 36/4 |
| Inhibition of matrix metalloproteases | 3.75E-07 | 19/32 (0.594) | 17/2 |
| Clathrin-mediated endocytosis signaling | 9.73E-06 | 50/149 (0.336) | 44/6 |
| CD28 signaling in T helper | 2.09E-05 | 38/106 (0.358) | 32/6 |
| Antigen presentation pathway | 6E-05 | 14/26 (0.538) | 14/0 |

^b^The functions and canonical pathways that were most significant to the data set were identified by Ingenuity Pathway Analysis.
