# Supplementary figures and images for: Quercetin suppresses immune cell accumulation and improves mitochondrial gene expression in adipose tissue of diet‐induced obese mice
Source: Mol Nutr Food Res. 2015 Nov 24;60(2):300–12. doi: 10.1002/mnfr.201500595 (PMC5063128; doi:10.1002/mnfr.201500595)

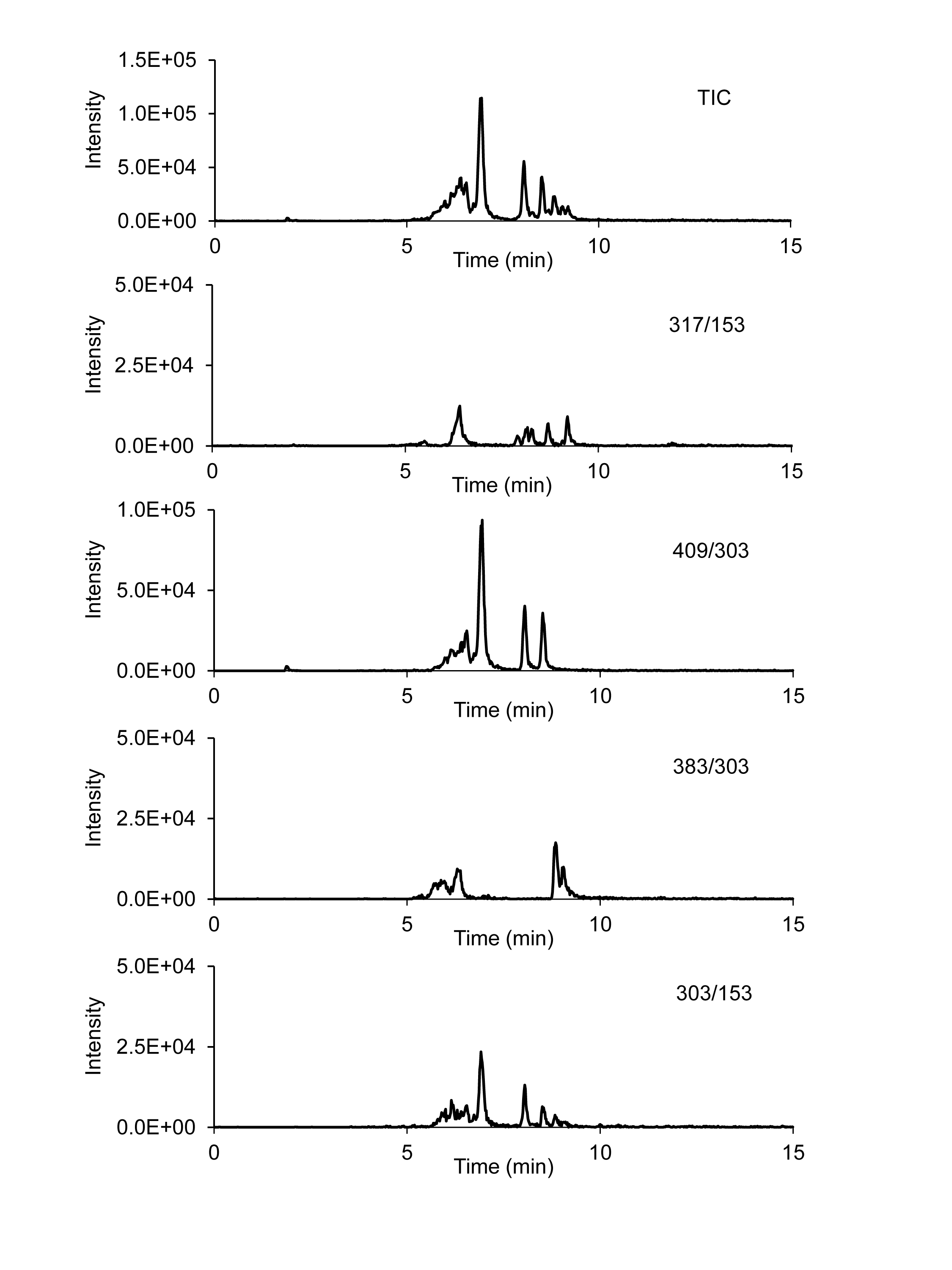

Supplement: Supplementary file 1 — Supplementary Material [file MNFR-60-300-s001.zip › mnfr2519-sup-0001-FigureS1.tif]
